# Urethrectomy with glans reconstruction and meatoplasty for rare high-grade urothelial carcinoma in distal urethra

**DOI:** 10.1016/j.eucr.2025.103270

**Published:** 2025-11-11

**Authors:** Mahima Gurushankar, Meher Pandher, Courtney Berg, Sandip M. Prasad, Amjad Alwaal

**Affiliations:** a Division of Urology, Department of Surgery, Rutgers New Jersey Medical School, Newark, NJ, USA; b Division of Urology, Department of Surgery, Morristown Medical Center, Morristown, NJ, USA

## Abstract

Urethral cancer is rare, with urothelial, squamous, and adenocarcinoma as the main histological subtypes. Distal urethral cancers are typically squamous, while urothelial carcinoma (UC) in this region is uncommon. We report a man who developed high-grade UC of the distal urethra eight years after excision of a prior squamous cell carcinoma (SCC) at the same site—to our knowledge the first reported case of sequential SCC and UC in this location. He underwent partial glansectomy and distal urethrectomy with reconstruction, revealing invasive UC with lymphovascular invasion and negative margins. This case highlights the need for long-term surveillance after urethral malignancy.

## 1 Introduction

Primary urethral carcinoma is a malignant tumor originating from urethral epithelium. The distal third of the urethra is lined with squamous epithelium, while the proximal two-thirds are composed of transitional epithelium. Urethral carcinomas are more often found in the proximal urethra than distal.^1^ The incidence of urethral carcinoma is highest in individuals aged ≥75 years, with rates increasing progressively with age.^2^^,^^3^ There are three primary histologic subtypes: urothelial (transitional cell) carcinoma (UC), squamous cell carcinoma (SCC), and adenocarcinoma, listed in order of prevalence.^2^^,^^4^ Urethral carcinoma is more common in males than females and occurs more frequently in African Americans compared to Caucasians.^2^^,^^3^^,^^5^ The 5-year relative survival rate for urethral cancer is approximately 54 %, with survival for carcinoma of the bulbar urethra reported as low as 20 %–30 %.^2^^,^^3^^,^^6^ Treatment for localized urethral cancer often involves penile-preserving surgical approaches, such as urethrectomy or partial penectomy, as well as radiation therapy.^1^^,^^5^^,^^7^, ^8^, ^9^ However, recurrence rates remain significantly high.^6^^,^^7^ For invasive urethral carcinomas in advanced stages, multimodal treatment is frequently necessary.^5^

Primary urethral malignancy is a rare condition in the United States, accounting for less than 1 % of all urogenital tumors.^10^ Significant predisposing factors include urethral strictures, lichen sclerosus, and urethral dilation.^11^ Similar to other cancers, the development of urethral carcinoma is strongly influenced by an inflammatory microenvironment.^5^^,^^12^ Identified risk factors include infection with oncogenic human papillomavirus (HPV) type 16, prior urethral procedures such as catheterization and urethroplasty, and radiation therapies for prostate cancer, including external beam radiotherapy and brachytherapy.^6^^,^^13^, ^14^, ^15^, ^16^, ^17^ Postoperative symptoms, such as lower urinary tract dysfunction or evidence of fistula formation, may signal recurrence. Providers should maintain a high index of suspicion to facilitate early diagnosis and timely intervention.^16^

We present the case of a 61-year-old male diagnosed with high-grade, invasive urothelial carcinoma originating from the distal urethral lining. This case is notable as the carcinoma arises from the distal urethra, a region typically associated with squamous cell carcinoma which the patient had 8 years prior. Due to the rarity of urethral carcinoma, the existing literature primarily comprises case reports and small retrospective analyses, as the low incidence precludes large-scale studies.^5^ This case contributes to the body of knowledge on transitional cell urethral carcinoma by detailing the patient's clinical presentation, treatment approach, and post-treatment outcomes.

## 2 Case presentation

The patient is a 61-year-old Caucasian male with a medical history notable for asthma and benign prostatic hyperplasia (BPH). His surgical history includes tonsillectomy, right knee surgery, nasal surgery, and appendectomy. He has documented allergies to penicillin, codeine, and trimethoprim-sulfamethoxazole. The only reported family history is maternal cancer, with the specific type and age of onset remaining unknown.

In December 2015, the patient presented with a mass lesion on the glans penis accompanied by microhematuria. He has no history of smoking or smokeless tobacco use and reports occasional social alcohol consumption, not occurring on a weekly basis. He denies illicit drug use and was not sexually active at the time of presentation. A review of systems was unremarkable, and his body mass index (BMI) was 24.64.

**Fig. 1:**
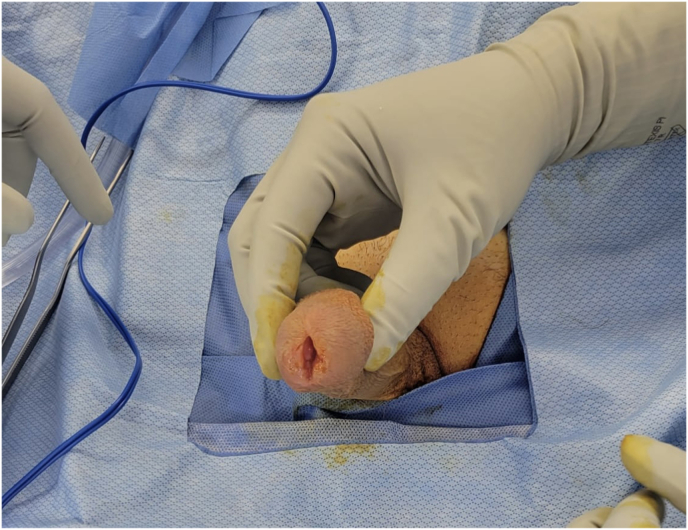
Preoperative photograph.

The patient reported that the mass had been present for approximately three years. Initial evaluations, including cystoscopy, FISH/cytology, and a sexually transmitted disease (STD) panel, were unremarkable. A computed tomography (CT) scan revealed nonspecific inguinal and pelvic lymphadenopathy, measuring up to 1.5 cm. In January 2016, an excisional biopsy performed at an outside hospital revealed pT2 SCC of the glans penis with invasion into the corpus spongiosum. Surgical margins were negative. The patient recovered well postoperatively, experiencing only mild urinary stream spraying. A follow-up CT scan one month after the excision demonstrated resolution of his lymphadenopathy, thus eliminating the need of a confirmatory biopsy. However, the patient developed urethral strictures following the procedure, which necessitated meatal dilation. The patient remained under surveillance and in July 2024, he developed recurrent ulceration at the urethral meatus (Fig. 1: preoperative photograph).

**Fig. 2:**
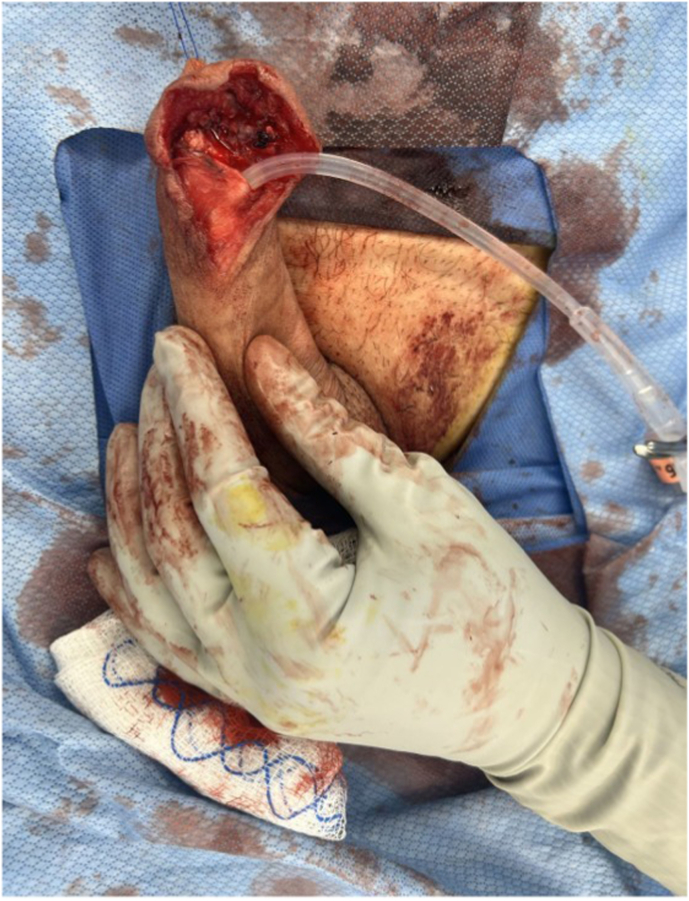
Partial glansectomy and distal urethrectomy.

**Fig. 3:**
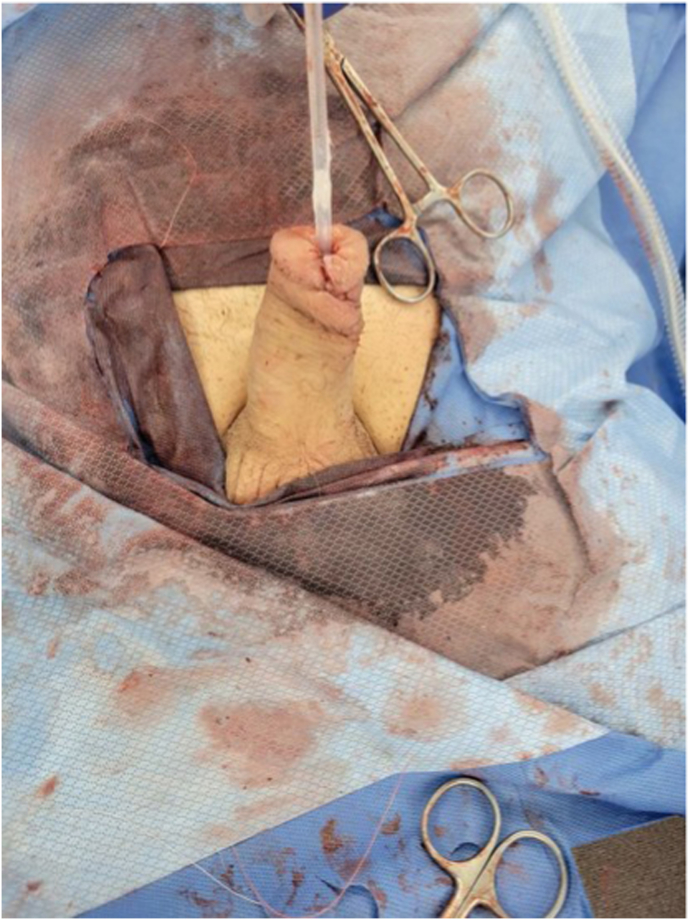
Glans reconstruction and meatoplasty after distal urethrectomy.

In August 2024, surgical exploration revealed a right-sided mass in the glans penis extending beyond the skin into the distal urethra. Initially, a partial penectomy was recommended but the patient was concerned about loss of erections. The patient underwent a partial glansectomy, distal urethrectomy, and subsequent urethral and glans reconstruction with meatoplasty (Fig. 2: partial glansectomy and distal urethrectomy; Fig. 3: glans reconstruction and meatoplasty after distal urethrectomy). Histopathological evaluation revealed pT1 invasive high-grade UC originating from the urethral lining with evidence of lymphovascular invasion. The urethral margins were free of malignancy.

Given his HGT1 UC, NCCN guidelines were reviewed but use of intraurethral BCG or chemotherapy appeared to be limited for this distal lesion. A CT urogram was recommended for upper tract evaluation along with surveillance cystoscopy every three months to monitor for recurrence. The patient was also referred for urine cytology, culture, and fluorescence in situ hybridization (FISH) prior to cystoscopy. At 4 months post-op, postoperative imaging with CT scan have demonstrated no suspicious lymphadenopathy. Follow-up cystoscopies have shown no evidence of urethral or bladder lesions.

The patient's postoperative recovery was unremarkable, with good healing observed and no evidence of stenosis with preservation of normal erections. The patient developed a small wound dehiscence at the distal shaft which healed by secondary intention. He voids without difficulty and without urethral dilation.

## 3 Discussion

Distal urethral carcinoma is commonly SCC due to the distal urethra being lined with squamous epithelial cells. This is similar to lesions arising from the glans penis and foreskin.^1^^,^^9^ These carcinomas are predominantly associated with human papillomavirus (HPV) infection.^9^ Although less common than SCC, UC has been rarely reported in the distal urethra.^8^^,^^18^ We believe that our case may be the first in the literature to describe UC recurrence following initial SCC in the distal urethra.

Presenting symptoms of UC include visible distal penile lesions, lower urinary tract symptoms (LUTS), hematuria, discharge, urinary obstruction, penile mass, fistula, bleeding, or metastasis, typically to inguinal lymph nodes.^9^^,^^18^^,^^19^ In this case, our patient presented originally with squamous cell carcinoma of the urethra detected after his primary surgery, and recurrence of a penile lesion led to detection of transitional cell carcinoma of the distal urethra.

Existing literature suggests that inflammatory procedures, such as catheterization and urethroplasty, may increase the risk of urethral carcinoma^6^^,^^14^^,^^16^

In a prior case of distal urethral TCC, a patient who presented with hematuria, a narrow urinary stream, and a palpable tumor was successfully treated with transurethral electro resection in 2010.^19^ Although postoperative negative margins indicate successful surgical removal of the tumor, another potential treatment modality for distal urethral TCC is radiation therapy.^8^ However, there is evidence suggesting that radiation therapy, when used in genital-preserving treatments, may be inadequate due to recurrence and lower survival rates.^20^ Given the rarity of urethral carcinoma, a standardized treatment protocol has yet to be established.^8^

Early detection and vigilant surveillance are essential in managing urethral malignancies due to their high-risk nature and propensity for recurrence. Although urethral carcinoma is a rare condition, particularly in the distal urethra, it is imperative to include this diagnosis in the differential when evaluating lesions near the meatus or in patients presenting with high-risk symptoms such as lower urinary tract symptoms (LUTS), hematuria, fistula formation, or regional lymphadenopathy. Close monitoring is advised not only for patients with a history of treated urethral carcinoma but also for those who have undergone any surgical procedures or treatments resulting in urethral inflammation. Prompt pathological examination of suspicious lesions is critical, as demonstrated in this case, where a glans SCC and a urethral UC were both diagnosed. Although there is no standard regimen for treatment, as there are not enough cases to witness a significant superiority in treatment regimens, and this case is evidence that surgery may prove effective.

Limitations are inherent to case studies, as they focus on a single -patient experience without the ability to control for confounding variables, making it challenging to establish causality between risk factors, disease, and outcomes. Additionally, due to the rarity of UC arising in the distal urethra, there is insufficient data to draw robust comparisons or identify meaningful trends. The limited availability of evidence may fail to comprehensively capture the full spectrum of disease etiologies, clinical presentations, and treatment outcomes. Long-term follow-up is particularly critical in such cases to evaluate survival rates, recurrence rates, and post-treatment complications; however, patient attrition and loss to follow-up often hinder the ability to perform thorough longitudinal analyses.

## CRediT authorship contribution statement

**Mahima Gurushankar:** Writing – review & editing, Writing – original draft, Project administration, Data curation. **Meher Pandher:** Resources, Investigation. **Courtney Berg:** Writing – review & editing. **Sandip M. Prasad:** Investigation, Formal analysis, Data curation. **Amjad Alwaal:** Supervision, Resources, Project administration, Methodology, Investigation, Conceptualization.
